# Comparison of various vasopressin doses to epinephrine during cardiopulmonary resuscitation in asphyxiated neonatal piglets

**DOI:** 10.1038/s41390-023-02858-x

**Published:** 2023-11-08

**Authors:** Marwa Ramsie, Po-Yin Cheung, Tze-Fun Lee, Megan O’Reilly, Georg M. Schmölzer

**Affiliations:** 1 Centre for the Studies of Asphyxia and Resuscitation, Neonatal Research Unit, Royal Alexandra Hospital, Edmonton, AB Canada; 2 Department of Pediatrics, University of Alberta, Edmonton, AB Canada

## Abstract

**Background:**

Current neonatal resuscitation guidelines recommend epinephrine for cardiac arrest. Vasopressin might be an alternative during asphyxial cardiac arrest. We aimed to compare vasopressin and epinephrine on incidence and time to return of spontaneous circulation (ROSC) in asphyxiated newborn piglets.

**Design/methods:**

Newborn piglets (*n* = 8/group) were anesthetized, intubated, instrumented, and exposed to 30 min of normocapnic hypoxia, followed by asphyxia and asystolic cardiac arrest. Piglets were randomized to 0.2, 0.4, or 0.8IU/kg vasopressin, or 0.02 mg/kg epinephrine. Hemodynamic parameters were continuously measured.

**Results:**

Median (IQR) time to ROSC was 172(103–418)s, 157(100–413)s, 122(93–289)s, and 276(117–480)s for 0.2, 0.4, 0.8IU/kg vasopressin, and 0.02 mg/kg epinephrine groups, respectively (*p* = 0.59). The number of piglets that achieved ROSC was 6(75%), 6(75%), 7(88%), and 5(63%) for 0.2, 0.4, 0.8IU/kg vasopressin, and 0.02 mg/kg epinephrine, respectively (*p* = 0.94). The epinephrine group had a 60% (3/5) rate of post-ROSC survival compared to 83% (5/6), 83% (5/6), and 57% (4/7) in the 0.2, 0.4, and 0.8IU/kg vasopressin groups, respectively (*p* = 0.61).

**Conclusion:**

Time to and incidence of ROSC were not different between all vasopressin dosages and epinephrine. However, non-significantly lower time to ROSC and higher post-ROSC survival in vasopressin groups warrant further investigation.

**Impact:**

- Time to and incidence of ROSC were not statistically different between all vasopressin dosages and epinephrine.
- Non-significantly lower time to ROSC and higher post-ROSC survival in vasopressin-treated piglets.
- Overall poorer hemodynamic recovery following ROSC in epinephrine piglets compared to vasopressin groups.
- Human neonatal clinical trials examining the efficacy of vasopressin during asphyxial cardiac arrest will begin recruitment soon.

## Introduction

The International Liaison Committee on Resuscitation recommends epinephrine when a newborn’s heart rate remains <60 beats/minute despite chest compressions (CCs) and positive pressure ventilation (PPV) with 100% oxygen.^1^ Epinephrine possesses chronotropic, dromotropic, inotropic, and lusitropic effects and is an effective systemic vasoconstrictor; however, it also increases myocardial oxygen demand with reduced contractile response during respiratory and metabolic acidosis.^2–4^ Furthermore, its infrequent use and the inability to predict which newborns will require cardiopulmonary resuscitation (CPR), has led to a lack of clinical data on epinephrine.^5,6^

Vasopressin may be an alternative as it is a systematic vasoconstrictor and pulmonary vasodilator whose mechanism of action is unaffected by metabolic or respiratory acidosis.^4,7^ Vasopressin mediates smooth muscle contraction, thereby directly increasing systemic vascular resistance and, consequently, enhancing coronary artery perfusion.^8^ In adults with asystolic out-of-hospital cardiac arrest, vasopressin resulted in significantly higher rates of survival to hospital admission (29% vs. 20%, *p* = 0.02) and discharge (5% vs. 2%, *p* = 0.04) compared to epinephrine.^9^ Asphyxia resulting in asystole is one of the main cause of cardiac arrest in newborns, therefore vasopressin might be an alternative during neonatal CPR. Contradicting results about the effectiveness of vasopressin during neonatal CPR have been reported. McNamara et al., randomized 69 newborn piglets to 0.01 or 0.03 mg/kg epinephrine, 0.2 or 0.4IU/kg vasopressin, or saline (control) during CPR.^10^ Vasopressin with 0.4IU/kg (*n* = 9/10 (90%)) resulted in higher survival rate compared to 0.01 mg/kg epinephrine (*n* = 5/13 (36%); *p* = 0.006) and control (*n* = 5/12 (43%); *p* = 0.03), while there was no difference in survival between 0.2IU/kg vasopressin and 0.03 mg/kg epinephrine.^10^ Rawat et al., randomized 27 near-term lambs to either 0.03 mg/kg epinephrine or 0.4IU/kg vasopressin and reported no difference in time or number of lambs achieving ROSC [70% (7/10) with epinephrine by 8(2) minutes and 33% (3/9) vasopressin lambs by 13(6) minutes, no *p* value reported].^11^

We aimed to compare different dosages of vasopressin with epinephrine during neonatal CPR in our post-transitional model of neonatal asphyxia. Our hypothesis was that vasopressin compared to epinephrine would decrease the time to return of spontaneous circulation (ROSC) in asphyxiated neonatal piglets.

## Methods

**Fig. 1:**
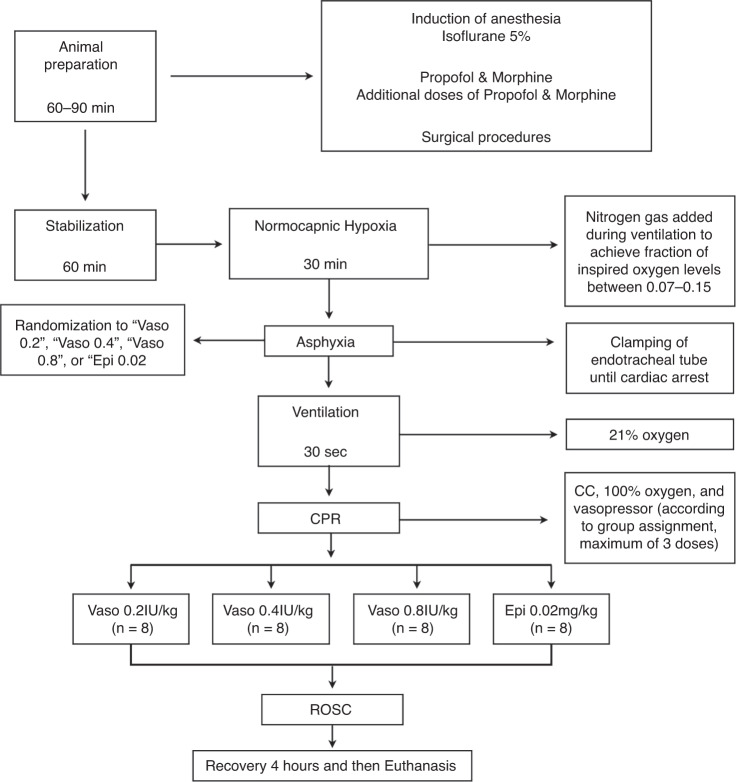
Study flow chart.

Thirty-two newborn mixed breed piglets were obtained on the day of experimentation from the University Swine Research Technology Center. Piglets were clinically normal and free of diseases that could affect results. All experiments were conducted in accordance with the guidelines and approval of the Animal Care and Use Committee (Health Sciences), University of Alberta [AUP00002920], presented according to the ARRIVE guidelines,^12^ and registered at preclinicaltrials.eu (PCTE0000378). A graphical display of the study protocol is presented in Fig. 1.

### Randomization

Piglets were randomly allocated to intravenous administration of vasopressin 0.2IU/kg, 0.4IU/kg, 0.8IU/kg, or epinephrine 0.02 mg/kg. Allocation was block randomized with variable sized blocks using a computer-generated randomization program (http://www.randomizer.org). Sequentially numbered, sealed, brown envelopes containing the allocation were opened during the experiment (Fig. 1).

### Blinding

GMS assessed cardiac arrest and was blinded to group allocation until confirmed cardiac arrest. Following stabilization, TFL opened the randomization envelope and was solely responsible for preparing and administering vasopressin/epinephrine. As TFL was not blinded to treatment allocation, he had no role in asphyxiation, cardiac arrest assessment, or administration of PPV. The content of the drug syringe was only known to TFL, the remaining team was blinded to the type of drug treatments.

### Animal preparation

Piglets were instrumented as previously described with modifications.^13–15^ Following the induction of anesthesia using isoflurane, piglets were intubated via a tracheostomy, and pressure-controlled ventilation (Sechrist Infant Ventilator Model IV-100; Sechrist Industries, Anaheim, California) was commenced at a respiratory rate of 16–20 breaths/min and pressure of 20/5cmH_2_O. Oxygen saturation was kept within 90–100%, glucose level and hydration was maintained with an intravenous infusion of 5% dextrose at 10 mL/kg/hr. During the experiment anesthesia was maintained with intravenous propofol 5–10 mg/kg/hr and morphine 0.1 mg/kg/hr. Additional doses of propofol (1–2 mg/kg) and morphine (0.05–0.1 mg/kg) were also given as needed. The piglet’s normothermic body temperature was maintained at 38.5–39.5 °C using an overhead warmer and a heating pad.

### Hemodynamic parameters

A 5-French Argyle^®^ (Klein-Baker Medical Inc. San Antonio, TX) double-lumen catheter was inserted via the right femoral vein for administration of fluids and medications. A 5-French Argyle^®^ single-lumen catheter was inserted above the right renal artery via the femoral artery for continuous arterial blood pressure monitoring in addition to arterial blood gas measurements. The right common carotid artery was also exposed and encircled with a real-time ultrasonic flow probe (2 mm; Transonic Systems Inc., Ithica, NY) to measure cerebral blood flow.

Piglets were placed in supine position and allowed to recover from surgical instrumentation until baseline hemodynamic measures were stable (minimum one hour). Ventilator rate was adjusted to keep the partial arterial CO_2_ between 35 and 45 mmHg as determined by periodic arterial blood gas analysis. Mean systemic arterial pressure (MAP), systemic systolic and diastolic arterial pressure, heart rate, and percutaneous oxygen saturation were continuously measured and recorded throughout the experiment with a Hewlett Packard 78833B monitor (Hewlett Packard Co., Palo Alto, CA).

### Cerebral perfusion

Cerebral regional oxygenation (crSO_2_) was measured using the Invos^TM^ Cerebral/Somatic Oximeter Monitor (Invos 5100, Somanetics Corp., Troy, MI). The sensors were placed on the right forehead of the piglet and secured with wrap and tape. Light shielding was achieved with a slim cap. The Invos^TM^ Cerebral/Somatic Oximeter Monitor calculates crSO_2_, which is expressed as the percentage of oxygenated hemoglobin (oxygenated hemoglobin/total hemoglobin). Values of regional oxygen saturation are stored every second with a sample rate of 0.13 Hz.^16^

### Experimental protocol

Following surgical instrumentation and stabilization procedure, a subsequently numbered, sealed brown envelope containing the assigned intervention was opened (Fig. 1). Piglets were randomized to receive an intravenous bolus of 0.1 mL/kg of one of the following four groups: 0.2IU/kg, 0.4IU/kg, 0.8IU/kg vasopressin, or 0.02 mg/kg epinephrine, followed with a 3 mL saline bolus. Piglets were exposed to 30 min of normocapnic hypoxia, followed by asphyxia. Asphyxia was achieved by disconnecting the ventilator and clamping the endotracheal tube until asystole. Asystole was defined as no heart rate audible during auscultation with a MAP ~ 0 mmHg, and the absence of carotid blood flow and discernable ECG activity. Fifteen seconds after diagnosed asystole, PPV was performed for 30 s with a Neopuff T-Piece (Fisher & Paykel, Auckland, New Zealand). The default settings of the experiment were a peak inflating pressure of 30cmH_2_O, a positive end expiratory pressure of 5cmH_2_O, and a gas flow of 8 L/min.

After 30 s of PPV, chest compressions (CCs) with sustained inflations were started. 100% oxygen was commenced 30 s after start of CCs. CCs were performed mechanically using an automated machine, specifically designed in our laboratory,^17–21^ at a rate of 90/min, acceleration of compression of 500 cm/s^2^, recoil speed of 50 cm/s, and with an anterior-posterior chest diameter of 33%. Vasopressin or epinephrine was administered intravenously 1 min after the start of CCs and administered every 3 min as needed if no ROSC was observed, to a maximum of three doses. ROSC was defined as an unassisted heart rate ≥100 bpm for 15 s. Successful ROSC was assessed by ECG activity and confirmed through auscultation by GMS. After ROSC, piglets recovered for four hours before the piglets were euthanized with an intravenous overdose of sodium pentobarbital (120 mg/kg). During the four hour post-ROSC period, piglets with a combined heart rate <50 bpm and MAP < 15 mmHg were defined as dead.

### Sample size and power estimates

Our primary outcome measure was incidence and time to achieve ROSC as well as hemodynamic changes. We hypothesized that the use of 0.8IU/kg vasopressin during CPR would reduce time to achieve ROSC. A sample size of 32 piglets (8 per group) was sufficient to detect a clinically important (20%) reduction in time to achieve ROSC (i.e., 180 s vs. 140 s), with 80% power and a 2-tailed alpha error of 0.05. We used 0.02 mg/kg of epinephrine and 0.2 IU/kg, 0.4 IU/kg, and 0.8 IU/kg of vasopressin as these doses were currently or previously recommended.^22,23^

### Data collection and analysis

Demographics of study piglets were recorded. Transonic flow probes, heart rate and pressure transducer outputs were digitized and recorded with LabChart^®^ programming software (ADInstruments, Houston, TX). Airway pressures, gas flow, tidal volume, and end tidal-CO_2_ were measured and analyzed using Flow Tool Physiologic Waveform Viewer (Philips Healthcare, Wallingford, CT).

The data are presented as mean (standard deviation–SD) for normally distributed continuous variables and median (interquartile range–IQR) when the distribution was skewed. For all respiratory parameters, continuous values during CPR were analyzed. The data was tested for normality (Shapiro-Wilk and Kolmogorov-Smirnov test) and compared using ANOVA for repeated measures using Tukey post-test. Fisher’s exact test was used for categorical variables. *P* values are 2-sided and *p* < 0.05 was considered statistically significant. Statistical analyses were performed with SigmaPlot (Systat Software Inc, San Jose).

## Results

Thirty-two newborn mixed breed piglets (0–3 days old, weight 1.8–2.4 kg) were obtained on the day of the experiment and there were no differences in the baseline parameters between the groups (Table 1). Respiratory parameters during chest compressions were not different between the groups (Table 2).

**Table 1:** Baseline characteristics.

	Vasopressin 0.2IU/kg (*n* = 8)	Vasopressin 0.4IU/kg (*n* = 8)	Vasopressin 0.8IU/kg (*n* = 8)	Epinephrine 0.02 mg/kg (*n* = 8)	*P* value
Age (days)	1 (1–2)	1 (1–3)	1 (1–2)	2 (0–3)	0.75
Weight (kg)	1.9 (1.9–2.0)	2.0 (1.9–2.2)	2.1 (1.8–2.4)	2.1 (1.8–2.3)	0.61
Sex (male/female)	4/4	5/3	6/2	5/3	0.96
Heart rate (bpm)	152 (134–183)	156 (154–173)	158 (150–171)	158 (134–185)	0.91
MAP (mmHg)	66 (62–70)	66 (61–68)	65 (54–69)	59 (55–80)	0.82
Systolic pressure (mmHg)	82 (79–92)	85 (79–93)	85 (76–89)	83 (77–106)	0.70
Diastolic pressure (mmHg)	51 (49–53)	49 (47–52)	48 (40–54)	44 (41–60)	0.75
Carotid flow (mL/min)	34 (27–45)	30 (28–41)	37 (29–46)	36 (30–42)	0.69
Cerebral oxygenation (%)	44 (42–46)	40 (35–46)	45 (36–52)	43 (38–48)	0.73
pH	7.52 (7.45–7.53)	7.50 (7.44–7.55)	7.46 (7.39–7.52)	7.47 (7.44–7.58)	0.40
Base excess (mmol/L)	2.0 (−1–4.2)	2.5 (−1.1–4.8)	2.6 (−0.3 ~ 2.8)	−1.7 (−1.8–4.6)	0.86
PaCO_2_ (torr)	32 (32–35)	33 (32–35)	35 (33–40)	34 (31–35)	0.20
PaO_2_ (torr)	66 (59–71)	64 (61–70)	63 (48–73)	71(57–80)	0.17
Lactate (mmol/L)	3.1 (2.7–3.8)	3.8 (2.6–6.5)	2.7 (2.3–3.6)	3.1 (2.5–4.1)	0.61

Data are presented as median (IQR); MAP, mean arterial blood pressure.

**Table 2:** Summary of respiratory parameters during chest compressions.

	Epinephrine 0.02 mg/kg	Vasopressin 0.2IU/kg	Vasopressin 0.4IU/kg	Vasopressin 0.8IU/kg	*P* value
Ventilation rate (/min)	60 (5)	56 (5)	57 (6)	54 (12)	0.823
Peak Inflation Pressure (mmHg)	30.6 (0.1)	30.2 (1.6)	30.5 (0.9)	29.3 (2.4)	0.709
Positive End Expiratory Pressure (mmHg)	31.3 (0.4)	31.5 (1.3)	31.3 (1.1)	30.5 (1.5)	0.809
Peak Inflation Flow (L/min)	4.4 (0.5)	5.1 (0.5)	4.6 (0.8)	4.2 (1.3)	0.836
Peak Expiratory Flow (L/min)	−7.6 (1.6)	−8.2 (0.7)	−7.7 (1.2)	−7.2 (2.6)	0.290
Tidal volume (mL/kg)	6.8 (1.7)	9.5 (1.6)	8.8 (3)	7.7 (2.5)	0.535
End-Tidal CO_2_ (mmHg)	12 (2)	20 (6)	20 (5)	19 (4)	0.996

Data are presented as mean (SD).

### Resuscitation

Resuscitation characteristics are presented in Table 3. Median (IQR) time to ROSC appeared shorter in vasopressin piglets compared to the epinephrine group, but this did not reach significance (*p* = 0.59). ROSC was achieved in 6 (75%), 6 (75%), 7 (88%), and 5 (63%) piglets in the 0.2 IU/kg, 0.4 IU/kg, 0.8 IU/kg vasopressin, and 0.02 mg/kg epinephrine groups, respectively (*p* = 0.94). Rates of post-ROSC survival, defined as survival four hours after ROSC, at which point piglets were euthanized, were 83% (5/6), 83% (5/6), 57% (4/6), and 60% (3/5) in vasopressin 0.2, 0.4, 0.8 IU/kg, and epinephrine groups, respectively (*p* = 0.61).

**Table 3:** Summary of asphyxia, resuscitation, and survival of asphyxiated piglets.

		Vasopressin 0.2IU/kg	Vasopressin 0.4IU/kg	Vasopressin 0.8IU/kg	Epinephrine 0.02 mg/kg	*P* value
Asphyxia time (sec)		292 (261–443)	483 (325–582)	325 (206–578)	509 (308–600)	0.39
After asphyxiation	pH	6.73 (6.69–6.82)	6.74 (6.69–6.82)	6.77 (6.69–6.87)	6.74 (6.71–6.83)	0.87
	PaCO_2_ (torr)	89 (76–104)	85 (71–96)	78 (73–91)	77 (64–110)	0.82
	Base excess (mmol/L)	−23 (−25 to −22)	−24 (−265 to −21)	−22 (−27 to −20)	−23 (−27 to −21)	0.93
	Lactate (mmol/L)	20 (17–20)	19 (17–20)	20 (18–20)	18 (16–20)	0.71
Resuscitation	Doses (n)	1 (1–2.5)	1 (1–3)	1 (1–1.8)	2 (1–3)	0.68
Achieving ROSC ^†^		6 (75%)	6 (75%)	7 (88%)	5 (63%)	0.94
ROSC time (sec)		172 (103–418)	157 (100–413)	122 (93–289)	276 (117–480)	0.59
Survival after ROSC ^†^		5 (83%)	5 (83%)	4 (57%)	3 (60%)	0.61
Survival time (min)		240 (211–240)	240 (240–240)	240 (174–240)	240 (91–240)	0.47

Data are presented as median (IQR), unless indicated ^†^ *n* (%).

### Changes in Hemodynamic Parameters

**Fig. 2:**
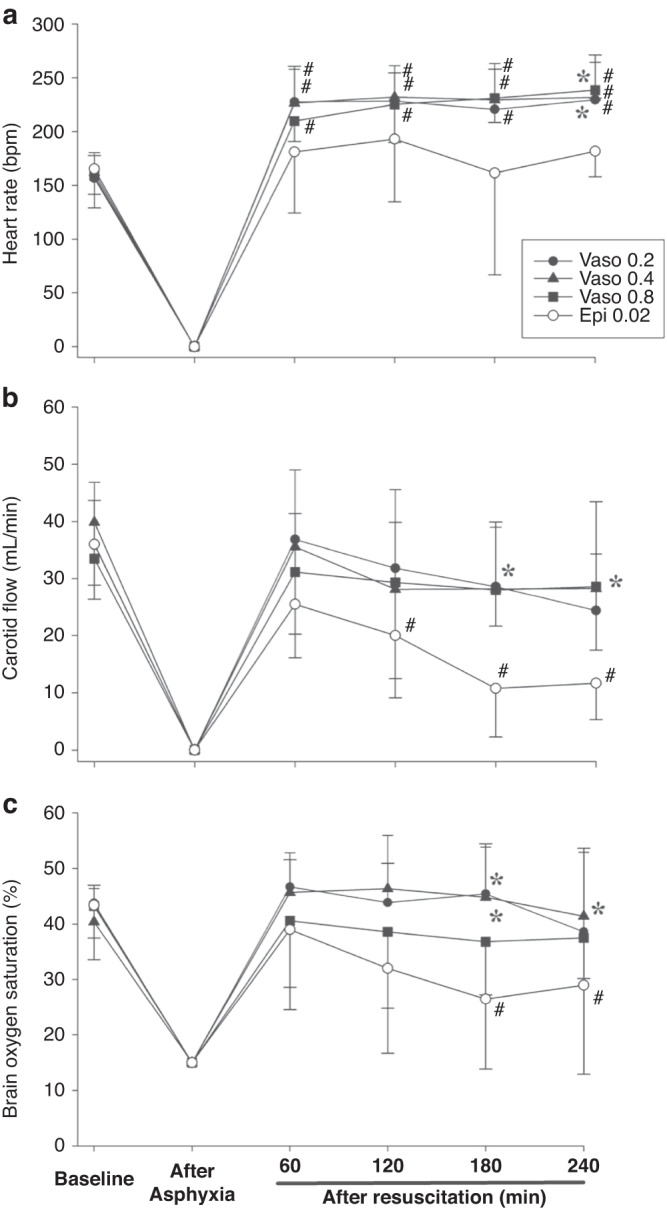
Changes in Heart rate, Carotid Blood Flow, and Brain Oxygenation. Hemodynamic changes in (**a**) heart rate (bpm, beats per minute), (**b**) carotid blood flow (mL/min), and (**c**) brain oxygen saturation (%) in piglets administered vasopressin 0.2IU/kg (closed circle), 0.4IU/kg (closed triangle), 0.8IU/kg (closed square), and 0.02 mg/kg epinephrine (open circle). Data are presented as mean (SD). # Significantly different from baseline; * Significantly different from the epinephrine 0.02 mg/kg group at the concurrent time point (*p* < 0.05).

**Fig. 3:**
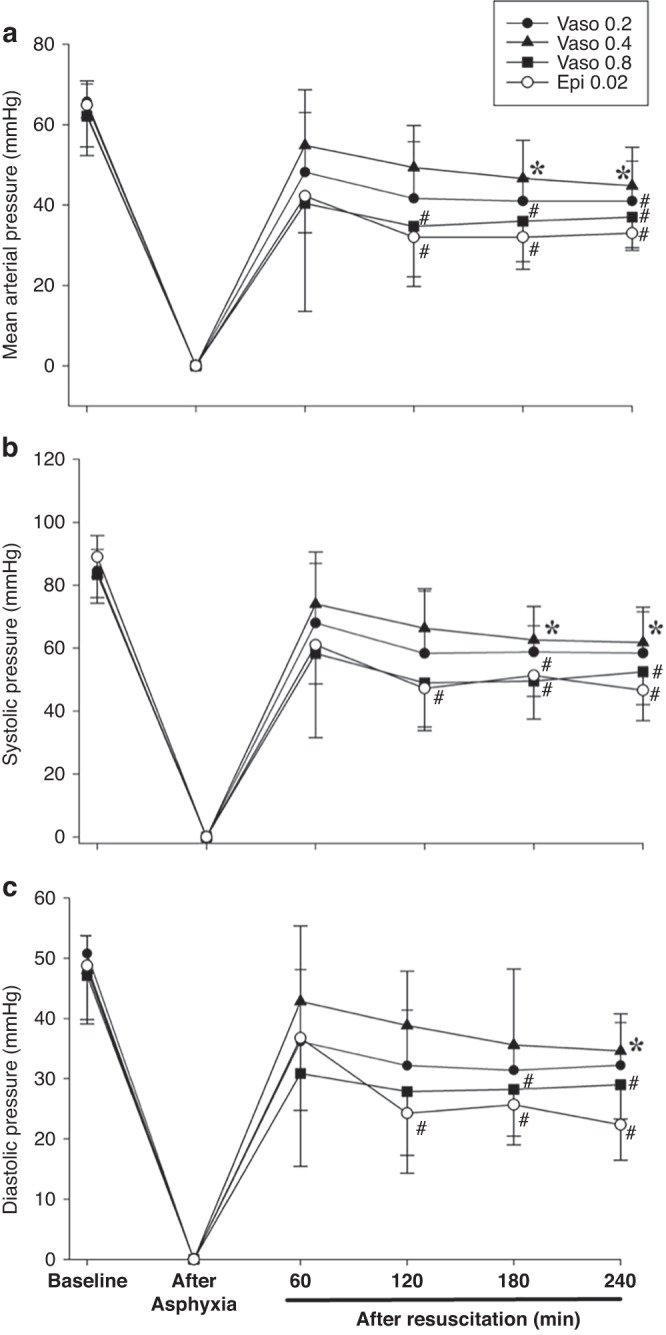
Changes in Mean Arterial, Systolic, and Diastolic Blood Pressure. Hemodynamic changes in (**a**) MAP, mean arterial pressure (mmHg), (**b**) systolic pressure (mmHg), and (**c**) diastolic pressure (mmHg) in piglets administered vasopressin 0.2IU/kg (closed circle), 0.4IU/kg (closed triangle), 0.8IU/kg (closed square), and 0.02 mg/kg epinephrine (open circle). Data are presented as mean (SD). # Significantly different from baseline; *Significantly different from the epinephrine 0.02 mg/kg group at the concurrent time point (*p* < 0.05).

Baseline hemodynamic parameters were not different between groups (Figs. 2, 3). Highest heart rates were observed in vasopressin 0.2IU/kg and 0.4IU/kg groups, while highest MAP was observed with vasopressin 0.4 IU/kg 240 min post-ROSC (*p* < 0.05). All hemodynamic changes of the epinephrine group, except heart rate, declined gradually throughout the recovery period and were significantly lower than baseline values by the end of the experimental period. Differences between vasopressin and 0.02 mg/kg epinephrine were not significantly different except for 240 min after resuscitation, in which heart rate, MAP, systolic and diastolic pressure, carotid blood flow, and cerebral regional oxygen saturation were all significantly higher with vasopressin 0.4IU/kg (*p* < 0.05). Changes in arterial blood gas are summarized in Table 4. Except for 0.8IU/kg, all vasopressin-treated piglets recovered back to their baseline values. In contrast, pH, base excess, and lactate were significantly lower than baseline in the epinephrine group 240 min after ROSC (*p* < 0.05).

**Table 4:** Arterial blood gas values in survivors before and after resuscitation.

	Vasopressin 0.2IU/kg	Vasopressin 0.4IU/kg	Vasopressin 0.8IU/kg	Epinephrine 0.02 mg/kg
pH				
Baseline	7.52 (7.45–7.53)	7.50 (7.44–7.55)	7.46 (7.39–7.52)	7.47 (7.44–7.58)
After asphyxiation	6.73 (6.69–6.82)^b^	6.74 (6.69–6.82)^b^	6.77 (6.69–6.87)^b^	6.74 (6.71–6.83)^b^
1 h after resuscitation	7.19 (7.07–7.30)^b^	7.28 (6.99–7.31)^b^	7.21 (6.81–7.28)^b^	7.08 (6.92–7.12)^b^
4 h after resuscitation	7.24 (7.22–7.38)^b^	7.38 (7.25–7.39)	7.36 (7.32–7.43)	7.11 (7.01–7.38)^b^
PaCO_2_ (torr)				
Baseline	32 (32–35)	33 (32–35)	35 (33–40)	34 (31–35)
After asphyxiation	89 (76–104)^b^	85 (71–96)^b^	78 (73–91)^b^	77 (64–110)^b^
1 h after resuscitation	34 (27–42)	33 (29–36)	36 (31–38)	34 (32–40)
4 h after resuscitation	42 (35–44)	37 (34–38)	34 (33–44)	37 (32–52)
Base excess (mmol/L)				
Baseline	2.0 (−1–4.2)	2.5 (−1.1–4.8)	2.6 (−0.3–2.8)	−1.7 (−1.8–4.6)
After asphyxiation	−23 (−25 to −22)^b^	−24 (−265 to −21)^b^	−22 (−27 to −20)^b^	−23 (−27 to −21)^b^
1 h after resuscitation	−16 (−19 to −13)^b^	−16 (−23 to −11)^b^	−15 (−29 to −11)^b^	−21 (−26 to −19)^b^
4 h after resuscitation	−7.6 (−13 to −1.8)^b^	−3.9 (−7.4 to −1.2)^a^	−3.9 (−12 to −1.4)^b^	−18 (−18 to −6.3)^b^
Lactate (mmol/L)				
Baseline	3.1 (2.7–3.8)	3.8 (2.6–6.5)	2.7 (2.3–3.6)	3.1 (2.5–4.1)
After asphyxiation	20 (17–20)^b^	19 (17–20)^b^	20 (18–20)^b^	18 (16–20)^b^
1 h after resuscitation	16 (14–20)^b^	17 (13–20)^b^	17 (14–20)^b^	20 (18–20)^b^
4 h after resuscitation	6.4 (3.4–12)	4.7 (4.6–6.7)^a^	6.2 (4.0–19)^b^	14.2 (5.6–16.7)^b^

Data are presented as median (IQR).

^a^Significantly different from epinephrine 0.02 mg/kg group.

^b^Significantly different from baseline values.

## Discussion

Current neonatal resuscitation guidelines recommend 0.01–0.03 mg/kg intravenous epinephrine during CPR if the heart rate has not increased to ≥60 beats/minute following PPV with 100% oxygen and CCs.^1,24^ However, epinephrine increases myocardial oxygen demand, potentially causing brain damage with persisting hypoxia, and can result in necrotizing enterocolitis or renal failure at high doses.^25,26^ Vasopressin may be an alternative to epinephrine; pediatric and adult studies reported that vasopressin is more effective during asystolic cardiac arrest, one of the main cardiac arrest rhythm in newborn infants.^9,27,28^ The current study compared three different vasopressin dosages with 0.02 mg/kg epinephrine in a neonatal post-transitional piglet model of asphyxial cardiac arrest. The results can be summarized as follows: (1) time to ROSC and survival was not statistically significant different between groups (Table 3); (2) rates of post-ROSC survival observed in vasopressin 0.2 and 0.4 IU/kg was 83% ((5/6) in both groups), 57% (4/7) in vasopressin 0.8 IU/kg, and 60% (3/5) in the epinephrine group (*p* = 0.61); and (3) overall hemodynamic and blood gas recovery of 0.4IU/kg vasopressin piglets was significantly better to that of the epinephrine group.

McNamara et al. demonstrated that treating piglets with vasopressin resulted in higher rates of survival compared to epinephrine (*p* < 0.05).^10^ However, Rawat et al., reported no difference in time to ROSC or the number of near-term lambs that achieved ROSC following treatment with either vasopressin or epinephrine.^11^ In the current study, rates of post-ROSC survival was 83% (5/6) in both 0.2 and 0.4IU/kg vasopressin groups and 60% (3/5) in the epinephrine group (Table 3; *p* = 0.61). Relatively improved post-ROSC survival in vasopressin groups may result from the ability of vasopressin to maintain its vasoconstricting efficacy during severe acidosis (pH < 7.2; effect on ɑ-adrenergic receptors but not vasopressin receptors), commonly present during asystole, whereas epinephrine does not.^4,29^

A rat model of asphyxial cardiac arrest compared 0.4, 0.8, and 2.4IU/kg intravenous vasopressin and saline control and reported all vasopressin-resuscitated rats had an 80% (8/10) rate of ROSC (*p* < 0.01), with mean(SD) times to ROSC of 60(17)s, 73(25) s, 84(44)s, and 205 s(no SD reported) in vasopressin 0.4, 0.8, 2.4 IU/kg, and saline groups, respectively (*p* > 0.05).^30^ Higher rates of ROSC reported by McNamara et al., and Chen et al., with vasopressin compared to epinephrine may be a result of improved systematic vasoconstriction with vasopressin.^4,31^ In the current study, median(IQR) time to ROSC was 122(93–289)s in the 0.8IU/kg vasopressin group, 157(100–413)s in the 0.4IU/kg vasopressin group, and 276(117–480)s in the epinephrine group (*p* = 0.59). As observed in the current study, Chen et al., reported heart rate was most improved in vasopressin 0.4IU/kg resuscitated rats following ROSC compared to all other groups (*p* < 0.05).^30^

Diastolic, systolic, and MAP with 0.4IU/kg vasopressin was significantly higher 240 min post-ROSC than the epinephrine group, which had significantly decreased values compared to baseline (Fig. 3; *p* < 0.05). Increased diastolic pressure 240 min after ROSC suggests improved coronary artery perfusion. Vasopressin and epinephrine half-lives are 10–35 and ~5 min, respectively.^32,33^ Therefore, hemodynamic differences 240 min post-ROSC are not a result of circulating vasopressor concentrations, and instead reflect patterns of recovery that persist following ROSC.

All hemodynamic changes, except heart rate, of the epinephrine group declined gradually throughout the recovery period and were significantly lower than baseline values by the end of the experimental period. Differences between vasopressin and 0.02 mg/kg epinephrine were not significantly different except 240 min after resuscitation, in which heart rate, MAP, systolic and diastolic pressure, carotid blood flow, and cerebral regional oxygen saturation were all significantly higher with vasopressin 0.4IU/kg (*p* < 0.05).

The vasopressin 0.4IU/kg group had significantly greater blood flow at 180- and 240-minutes post-ROSC compared to the epinephrine group (Fig. 2b); (*p* < 0.05), which had significantly lower blood flow than its baseline values at 120-, 180-, and 240-minutes following ROSC. Brain oxygen saturation values of vasopressin groups returned to baseline following ROSC at all time points, while the epinephrine group had significantly decreased saturation values at 180- and 240-minutes post-ROSC compared to baseline (Fig. 2c); (*p* < 0.05). Brain oxygen saturation was significantly higher in the 0.4IU/kg vasopressin group at 180- and 240-minutes post-ROSC compared to the epinephrine group (Fig. 2c); (*p* < 0.05). Decreased carotid blood flow and brain oxygen saturation in the epinephrine group following ROSC are likely a result of its ɑ_1_-agonist action, which decreases cerebral cortical microcirculatory blood flow and increases cerebral ischemia and hypercarbia following ROSC.^34^ Comparatively, vasopressin increases carotid blood flow, cerebral perfusion, and brain oxygen saturation.^35,36^ We speculate increased MAP following ROSC in vasopressin groups does not result in hyperperfusion injuries, as values returned to baseline.

Rawat et al., did not report a significant difference in carotid blood flow between epinephrine and vasopressin groups; however, stroke carotid flow volume, defined as carotid flow volume per heartbeat, was significantly higher in vasopressin lambs (0.175(0.08)mL/kg) compared to the epinephrine group (0.109(0.02)mL/kg; *p* < 0.001).^11^ The same study euthanized lambs approximately 20 min after ROSC, therefore potentially significant differences between vasopressin and epinephrine groups that may exist beyond the immediate post-ROSC period were not reported.^11^ Rawat et al., reported increased pulmonary blood flow in vasopressin-resuscitated lambs compared to the epinephrine group (*p* = 0.0001), while McNamara et al., reported a decreased inverse ratio of pulmonary artery acceleration time to right ventricular ejection in the vasopressin groups, indicating lower pulmonary vascular resistance in the vasopressin groups.^10,11^ Rapid restoration of cerebral blood flow and brain oxygen saturation to baseline values with 0.4IU/kg vasopressin in the current study may translate to favorable neurological outcomes following ROSC. Clinical trials comparing vasopressin with epinephrine during CPR including neurological outcomes are warranted. Few adverse effects of vasopressin have been documented. Vasopressin decreased cardiac output in adult dogs with pulmonary hypertension with an infusion of high dose vasopressin (1.16IU/kg/hour; *p* < 0.05).^37^ Additionally, cases of intestinal ischemia have been reported in critically ill patients following high single doses (~4–16IU) of vasopressin.^38^ Increased digital ischemia and severe diarrhea has been reported in septic shock patients treated with high-dose terlipressin (4 mg/day), a synthetic analog of vasopressin.^39^

## Limitations

The use of an automated CC machine reduces variations that may arise from human-administered CCs. However, several limitations should be considered before implementing vasopressin in neonatal resuscitation. Our asphyxia model uses piglets that have already undergone the fetal-to-neonatal transition, and piglets were sedated/anesthetized. Furthermore, our model requires piglets to be intubated with a tightly sealed endotracheal tube to prevent any endotracheal tube leak; this may not occur in the delivery room as mask ventilation is frequently used. Nevertheless, our findings are still clinically relevant as the distribution of cardiac output to vital organs (i.e. brain and heart) in the fetus and post-transitional neonate during asphyxia episodes are quantitatively similar.^40,41^ Our resuscitation model is slightly different from the currently recommended resuscitation guidelines, as we administered epinephrine 90 s after PPV initiation. This may have influenced our results. Additionally, piglets were euthanized four hours after ROSC; therefore, no comparisons could be made on long-term outcomes between groups, which is a limitation of this study.

## Conclusion

Time to and incidence of ROSC were not different between all vasopressin dosages and epinephrine. However, better hemodynamic and blood gas recoveries following asphyxia-induced asystolic cardiac arrest in the 0.4IU/kg vasopressin group suggests this may be the optimal dosage during neonatal resuscitation. Improved hemodynamic parameters following ROSC with 0.4IU/kg vasopressin compared to epinephrine warrants further investigation.

### Data sharing

All data generated or analyzed during this study are included in this published article. Data used to generate the results reported in this study will be made available following publication to researchers who provide a methodologically sound proposal.

### Supplementary information

ARRIVE_checklist
